# Comparison of discectomy with and without fusion in the surgical treatment of recurrent lumbar disc herniation

**DOI:** 10.1007/s10143-025-03687-8

**Published:** 2025-07-05

**Authors:** Ali Osman Mucuoglu, Huseyin Dogu, Hidayet Akdemir

**Affiliations:** School of Medicine, Department of Neurosurgery, Atlas University, Istanbul, Turkey

**Keywords:** Recurrent lumbar disc herniation, Revision microdiscectomy, Posterolateral fusion, Interbody fusion

## Abstract

**Objective:**

Recurrent intervertebral disc herniation is one of the most common problems encountered in spine surgery. This study aimed to compare the clinical outcomes of revision microdiscectomy and fusion surgeries in patients with recurrent lumbar disc herniation.

**Methods:**

276 patients who underwent surgery with same surgeon for recurrent lumbar disc herniation between January 2012 and December 2023 were retrospectively analyzed. The patients were divided into three groups: Group 1 (revision microdiscectomy, *n* = 129), Group 2 (discectomy with posterolateral fusion, *n* = 123), and Group 3 (discectomy with posterolateral fusion and posterior lumbar interbody fusion, *n* = 24). Clinical outcomes were evaluated using pre- and postoperative VAS and JOA scores.

**Results:**

Postoperative radicular and lumbar VAS scores were significantly higher in Group 1 compared with Groups 2 and 3. The recovery rates were highest in Group 3 (77%) and Group 2 (75.5%), while Group 1 showed a lower recovery rate (71.8%). Postoperative JOA scores improved significantly in all groups, with Group 3 showing the greatest improvement in total JOA scores and SLR test results.

**Discussion:**

Fusion procedures were associated with better pain control and functional improvement but higher risks of complications, including screw malposition and cage displacement. Both revision microdiscectomy and fusion surgeries are effective for recurrent lumbar disc herniation. Revision microdiscectomy is effective in young patients with limited back pain but carries risks of recurrence and instability. Fusion surgeries provide superior pain relief and functional outcomes but carry higher risks of complications and longer recovery times.

**Conclusion:**

Both revision microdiscectomy and discectomy with fusion appear to be effective surgical options for the management of recurrent lumbar disc herniation. The selection of the surgical technique should be guided by patient-specific factors such as age and the predominance of low back versus radicular pain.

**Supplementary Information:**

The online version contains supplementary material available at 10.1007/s10143-025-03687-8.

## Introduction

Microdiscectomy remains the most widely utilized surgical approach for lumbar disc herniation (LDH) and is regarded as the gold standard [1]. Although microdiscectomy is considered comman techniques, recurrence is a leading factor that affects surgical outcomes. Despite its efficacy, recurrence poses a critical challenge impacting surgical outcomes. Recurrent disc herniation is defined as a new herniation at the same level, either ipsilateral or contralateral, after a pain-free interval of at least six months postoperatively [2]. Recurrence rates have been reported in the literature to range between 7% and 24% [3].

Recurrence is influenced by a variety of factors, with obesity, younger age, male gender, smoking, diabetes mellitus, and the size of the annular tear being the most significant [4]. Additionally, recent studies show that different surgical techniques are associated with varying recurrence rates, with recurrence being higher in limited discectomies compared to aggressive discectomies [3].

Currently, there is no consensus on the surgical technique of choice for recurrent lumbar disc herniation. Revision microdiscectomy is the most frequently preferred procedure in cases of recurrent disc herniation [5]. Otherwise, the outcomes of revision microdiscectomies may be worse than those of the primary surgery [6]. Disruption of the anatomical plan following the primary surgery and the risk of nerve and dura injury due to fibrosis tissue are the main reasons for the deterioration of clinical outcomes [7].

The guidelines prescribed by the American Association for Neurological Surgery in 2014 indicated that there was low-level evidence in support of posterior fusion in recurrent disc herniations and further research was recommended [8]. Furthermore, some previous studies have reported that posterior fusion cases have better pain control outcomes despite higher cost, higher blood loss, longer operative time, and longer duration of hospital stay [9].

Previous studies on surgical techniques for recurrent lumbar disc surgery, including revision microdiscectomy or fusion, and their respective results have been reported in the literature. Some advocated microdiscectomy only when radiculopathy is present [8]. Another group suggested fusion surgery in cases of lumbar instability, degenerative changes on radiologic images, and chronic low back pain [9]. Other studies have also recommended percutaneous endoscopic discectomy, a minimally invasive procedure, instead of revision microdiscectomy, which could lead to instability and complications, to avoid fusion surgery [7].

The present study aimed to compare the clinical outcomes of revision microdiscectomy and posterior fusion surgeries performed in recurrent lumbar disc cases with VAS and JOA scoring [4, 5].

## Materials and methods

In this study, we retrospectively analyzed 312 patients with recurrent herniated disc who underwent surgery with same surgeon in our clinic between January 2012 and December 2023 using information available in the patient files, radiologic data, and telephone questionnaires. Patients who were available for follow-up were evaluated during the outpatient clinic visits. Of these patients, 36 were excluded from the study because they could not be reached and the radiological images could not be examined. Therefore, in this study, 276 patients were included, each followed for a minimum of 18 months after surgery, with clinical outcome analysis conducted at the 12-month postoperative mark.

This study was approved by the local ethics committee with protocol number E-22686390-050.99-24887 informed consent was obtained from all patients before inclusion in the study.

### Preoperative evaluation

#### Inclusion criteria

1. Magnetic resonance imaging (MRI) of lumbosacral recurrent disc herniation at the same level as the first discectomy surgery.
2. Symptom-free for at least 6 months following primary lumbar disc surgery followed by recurrent low back pain with radiculopathy.
3. Received conservative treatment for at least 6 weeks and was ineffective.
4. Recurrent low back pain with progressive neurological deficit.

#### Exclusion criteria

1. Recurrent prolapse of the lumbar intervertebral disc at two or more levels.
2. Cauda equina syndrome.
3. Disc herniation with comorbid pathologies, such as infection, tumor, multisegmental spinal canal stenosis, disc herniation at the adjacent level, spondylolisthesis, and spinal deformities.

All demographic characteristics, including age, sex, pain-free time interval, side of re-herniation and surgical data, intra- and postoperative complications, visual analog score (VAS), and Japanese Orthopedic Association (JOA) scoring for pre- and postoperative pain of all patients were obtained from the medical records and evaluated.

All patients underwent a postoperative X-ray for control purposes, and CT and MRI were performed when deemed necessary.

The type of surgery was decided together with the patients after they were informed in detail about the method, results, and possible complications. Required consents for operation were collected.

### Groups

In this study, a total of same surgeon’s 276 patients were divided into 3 groups. Group 1 comprised 129 patients with recurrent disc herniation who underwent revision microdiscectomy. Group 2 comprised 123 patients who underwent discectomy with posterolateral fusion(PLF) with transpedicular screws. Group 3 comprised 24 patients who underwent discectomy, posterolateral fusion, and posterior lumbar interbody fusion (PLIF) with interbody cage.

### Surgical technique

All patients underwent surgery in the prone position under general anesthesia. A midline incision was made through the site of the previous classic incision. In Group 1, only revision microdiscectomy was performed. For Groups 2 and 3, transpedicular screws were placed first. In Group 3, an additional interbody fusion was performed, and autograft bone grafting were placed inside for fusion. In Group 2, autograft bone grafting was used for posterolateral fusion. During discectomy, the previous laminotomy site was expanded. Dissection was performed from the cranial side to access the disc space. However, in cases of severe adhesion, the dura and nerve root were exposed caudally from the upper part of the lower lamina and through the foramen to reach the disc space, where discectomy was performed. Anterior-posterior and lateral images were obtained using a fluoroscopy device, and the instrumentation was confirmed. Hemostasis was achieved. Hemovac drains were used in Groups 2 and 3. Patients who underwent revision microdiscectomy were mobilized without a brace, whereas those who underwent fusion were mobilized with a brace 8 h postoperatively. It was recommended that patients who underwent fusion wear a brace for 6 weeks during the postoperative period.

### Postoperative evaluation

#### Visual analog scale (VAS)

Patients were asked to rate their pain on a scale of 0–10, where “0” indicated no pain, and “10” indicated the most intolerable pain [5]. This number showed the degree of pain.

#### Japanese orthopedic association (JOA) scoring

1. Subjective Symptoms (total 9 points).

A. Lumbar pain: (a) None (3 points), (b) Sometimes (2 points), (c) Frequently (1 point), (d) Constant (0 points).
B. Leg pain: (a) None (3 points), (b) Intermittent mild pain (2 points), (c) Intermittent severe pain (1 point).
C. Always severe (0 points).
D. Walking: (a) Normal (3 points), (b) Walks more than 500 m, but with pain (2 points), (c) Walks less than 500 m (1 point), (d) Walks less than 100 m (0 points).

2. Clinical Findings (total 6 points).

A. Straight Leg Raise (SLR) Test: (a) Normal (2 points), (b) 30–70 degrees (1 point), (c) less than 30 degrees (0 points).
B. Sensory Damage: (a) None (2 points), (b) Mild sensory damage (1 point), (c) Significant sensory damage (0 points).
C. Motor Damage: (a) Normal muscle strength (2 points), (b) Mild weakness (1 point), (c) Significant weakness (0 points).

3. Restriction in Daily Living Activities (total 14 points).

A. Turning while lying down: Constant pain (2 points), infrequent pain (1 point), no pain (0 points).
B. Standing: Constant pain (2 points), infrequent pain (1 point), no pain (0 points).
C. Bathing: Constant pain (2 points), infrequent pain (1 point), no pain (0 points).
D. Lying down: Constant pain (2 points), infrequent pain (1 point), no pain (0 points).
E. Sitting: Constant pain (2 points), infrequent pain (1 point), no pain (0 points).
F. Lifting and holding: Constant pain (2 points), infrequent pain (1 point), no pain (0 points).
G. Walking: Constant pain (2 points), infrequent pain (1 point), no pain (0 points).

4. Bladder Function (Incontinence or urinary retention).

A. Normal (0 points).
B. Mild dysuria (− 3 points).
C. Severe dysuria (− 6 points).

Recovery Rate (%): (postoperative JOA score − preoperative JOA score) x 100/(29-preoperative JOA score).

Based on above formula, rate of success would be evaluated as very good for 90–100%, good for 70–90%, fair for 50–70%, and poor for 50% and below [4].

### Data evaluation

The Statistical Package for the Social Sciences (SPSS) ver. 26 software was used for statistical analyses in the study. Quantitative variables are presented as mean, standard deviation, median, and min–max values, whereas qualitative variables are expressed as descriptive statistical methods, including frequency and percentage. The Shapiro–Wilks test and box plots were used to test the normal distribution hypothesis for the study data.

One-way analysis of variance was used for quantitative two-group evaluations with a normal distribution, and Bonferroni’s test was used to determine the group that might have caused the difference.

The Wilcoxon signed-rank test was used for intragroup evaluations of variables that did not meet the normal distribution hypothesis, the Kruskal–Wallis test was used for the comparisons of three or more groups, and the Dunn test was used to determine the group that might have caused the difference.

The Chi-square test and Fisher–Freeman–Halton test were used to compare the qualitative data. The results were evaluated at a 95% confidence interval (CI), and a p value of 0.05 was considered statistically significant.

## Results

A total of 276 patients were included in the study, of which 43.5% (*n* = 120) were male and 56.5% (*n* = 156) were female. The age of the patients ranged from 28 to 87 years, with a mean age of 54.14 ± 11.88 years. Further, 50.7% (*n* = 140) and 49.3% (*n* = 136) had left- and right-sided herniation. The highest occurrence rate was observed at the L4-L5 level (60.5%, *n* = 167). The time of recurrence ranged from 6 to 121 months postoperatively, with a mean duration of 30.49 ± 21.14 months. Revision microdiscectomy (Group 1), discectomy + PLF (Group 2), and discectomy + PLF + PLIF (Group 3) groups included 46.7% (*n* = 129), 44.6% (*n* = 123), and 8.7% (*n* = 24), respectively.

Analysis of patients by group indicated statistically significant differences. Male patients are more likely to be included in the Group 1 than the Group 2. Patients included in the group 2 were older than those in the group 3. Patients in the group 2 were more likely to undergo surgery at the L4-L5 level. The time to recurrence was significantly higher in the group 2 compared with the group 3. (Table 1)

**Table 1:** Comparison of descriptive characteristics by groups

		Group 1 (*n* = 129)	Group 2 (*n* = 123)	Group 3 (*n* = 24)	*p*
**Sex**	**Male**	68 (52.7)	43 (35.0)	9 (37.5)	^***a***^ 0.015*
	**Female**	61 (47.3)	80 (65.0)	15 (62.5)	
**Age**	*Med ± STD*	53.77 ± 12,17	55.80 ± 11.22	47.71 ± 11.74	^***b***^ 0.008**
**Side**	**Left**	70 (54.3)	58 (47.2)	12 (50)	^***a***^ ***0.528***
	**Right**	59 (45.7)	65 (52.8)	12 (50)	
**Level**	**L1-L2**	1 (0.8)	1 (0.8)	0 (0)	^***c***^ 0.005**
	**L2-L3**	0 (0)	1 (0.8)	0 (0)	
	**L3-L4**	10 (7.8)	12 (9.8)	0 (0)	
	**L4-L5**	66 (51.2)	86 (69.9)	15 (62.5)	
	**L5-S1**	52 (40.3)	23 (18.7)	9 (37.5)	
**Recurrence (months)**	*Med ± STD*	28.79 ± 19.37	33.87 ± 23.82	22.25 ± 9.71	^***b***^ 0.022*

^a^Pearson’s Chi-Squared Test

^b^One-way ANOVA Test and Bonferroni Test

^c^Fisher–Freeman–Halton Test

**p < 0.01 *p < 0.05

### For VAS measurements

Postoperative radicular VAS scores were significantly higher in the group 1 than in the group 2 and group 3. The radicular VAS scores significantly decreased postoperatively of all three groups.

Postoperative lumbar VAS scores were significantly higher in the group 1 than in with the group 2 and group 3. Furthermore, the mean decrease in postoperative lumbar VAS scores of the three groups compared to preoperative values was statistically significant. The preoperative–postoperative change in VAS scores was significantly lower in the group 1 than in the group 2. (Table 2)

**Table 2:** VAS scores of the patients by groups

		Group 1 (*n* = 129)	Group 2 (*n* = 123)	Group 3 (*n* = 24)	Total	^d^ *p*
**Radicular VAS**						
**Preoperative**	*Med ± STD*	7.53 ± 1.11	7.56 ± 1.12	7.21 ± 1.18	7.52 ± 1.12	***0.379***
**Postoperative**	*Med ± STD*	2.02 ± 0.77	1.81 ± 0.82	1.58 ± 0.65	1.89 ± 0.79	0.008**
**Change ∆**	*Med ± STD*	-5.51 ± 1.19	-5.75 ± 1.24	-5.63 ± 1.13		***0.374***
**Low Back VAS**						
**Preoperative**	*Med ± STD*	7.36 ± 1.13	7.39 ± 1.13	6.71 ± 0.91	7.32 ± 1.12	0.009**
**Postoperative**	*Med ± STD*	2.16 ± 0.70	1.78 ± 0.68	1.58 ± 0.65	1.94 ± 0.72	0.001**
**Change ∆**	*Med ± STD*	-5.20 ± 1.18	-5.61 ± 1.23	-5.13 ± 0.80		0.019*

^d^Kruskal Wallis Test & Dunn-Bonferroni Test

^e^Wilcoxon Signed Rank Test

**p < 0.01 *p < 0.05

### For JOA measurements

Postoperative lumbar JOA scores were significantly lower in the group 1 compared to the group 2 and group 3. There was an increase in postoperative lumbar JOA scores compared to preoperative scores by groups, but there was no statistically significant difference between the groups.

A statistically significant difference was observed between the postoperative leg JOA scores of the patients by groups. Postoperative leg JOA scores were significantly lower in the group 1 compared to the group 2. A significant increase in postoperative leg JOA scores was also observed in each group. This increase was significantly higher in the group 2.

No statistically significant difference was observed between the postoperative walking JOA scores of the patients by groups. However, there was a significant increase in each group compared with the preoperative scores.

A statistically significant difference was observed between the postoperative SLR JOA scores of the patients by group. The postoperative SLR JOA scores of the group 1 were significantly lower compared to those of the group 2 and group 3. In all three groups, there was a statistically significant increase in postoperative SLR JOA scores compared to preoperative scores. This increase was significantly higher in the group 2 compared to the other groups.

There was no statistically significant difference between the postoperative sensory JOA scores of the patients by group. However, the increase in postoperative sensory JOA scores compared with preoperative scores was statistically significant in all three groups.

There was no statistically significant difference between the postoperative motor JOA scores of the patients by group. Nevertheless, the increase in postoperative motor JOA scores compared to preoperative was statistically significant in all the three groups.

There was no statistically significant difference between the postoperative activity JOA scores of the patients by group. Yet, the increase in postoperative activity JOA scores compared to preoperative was statistically significant in all the three groups. Furthermore, the amount of change in the group 3 was significantly higher compared to the group 1 and group 2.

There was no statistically significant difference between the postoperative bladder JOA scores of the patients by group. (Table 3)

**Table 3:** JOA scores of patients by groups

		Group 1 (*n* = 129)	Group 2 (*n* = 123)	Group 3 (*n* = 24)	Total	^d^ *p*	
Low Back JOA							
**Preoperative**	*Med ± STD*	1.52 ± 0.67	1.52 ± 0.68	1.5 ± 0.78	1.52 ± 0.68	***0.964***	
**Postoperative**	*Med ± STD*	2.50 ± 0.63	2.75 ± 0.51	2.79 ± 0.41	2.63 ± 0.57	0.001**	
**Change ∆**	*Med ± STD*	0.98 ± 0.90	1.23 ± 0.79	1.29 ± 0.81		***0.071***	
**Leg JOA**							
**Preoperative**	*Med ± STD*	1.25 ± 0.69	1.24 ± 0.69	1.21 ± 0.72	1.24 ± 0.69	***0.971***	
**Postoperative**	*Med ± STD*	2.51 ± 0.60	2.80 ± 0.44	2.79 ± 0.41	2.66 ± 0.54	0.001**	
**Change ∆**	*Med ± STD*	1.26 ± 0.87	1.56 ± 0.84	1.58 ± 0.93		0.040*	
**Walking JOA**							
**Preoperative**	*Med ± STD*	0.63 ± 0.49	0.62 ± 0.49	0.54 ± 0.51	0.62 ± 0.49	***0.727***	
**Postoperative**	*Med ± STD*	2.62 ± 0.56	2.7 ± 0.54	2.71 ± 0.55	2.66 ± 0.55	***0.334***	
**Change ∆**	*Med ± STD*	1.99 ± 0.72	2.08 ± 0.75	2.17 ± 0.76		***0.423***	
**SLR JOA**							
**Preoperative**	*Med ± STD*	0.68 ± 0.6	0.67 ± 0.61	0.75 ± 0.68	0.68 ± 0.61	***0.898***	
**Postoperative**	*Med ± STD*	1.66 ± 0.52	1.95 ± 0.22	1.88 ± 0.34	1.81 ± 0.42	0.001**	
**Change ∆**	*Med ± STD*	0.98 ± 0.80	1.28 ± 0.66	1.13 ± 0.74		0.012*	
**Sensory JOA**							
**Preoperative**	*Med ± STD*	0.76 ± 0.6	0.73 ± 0.59	0.75 ± 0.68	0.75 ± 0.6		***0.937***
**Postoperative**	*Med ± STD*	1.67 ± 0.49	1.76 ± 0.43	1.75 ± 0.44	1.72 ± 0.46		***0.317***
**Change ∆**	*Med ± STD*	0.91 ± 0.75	1.03 ± 0.56	1.00 ± 0.72			***0.588***
**Motor JOA**							
**Preoperative**	*Med ± STD*	1.36 ± 0.62	1.36 ± 0.63	1.42 ± 0.65	1.36 ± 0.63		***0.879***
**Postoperative**	*Med ± STD*	1.68 ± 0.48	1.67 ± 0.47	1.71 ± 0.46	1.68 ± 0.48		***0.896***
**Change ∆**	*Med ± STD*	0.33 ± 0.80	0.31 ± 0.53	0.29 ± 0.62			***0.978***
**Activity JOA**							
**Preoperative**	*Med ± STD*	6.04 ± 1.28	6.02 ± 1.27	5.13 ± 2.13	5.95 ± 1.39		***0.088***
**Postoperative**	*Med ± STD*	11.29 ± 1.4	11.58 ± 1.32	12.42 ± 1.72	11.52 ± 1.42		***0.065***
**Change ∆**	*Med ± STD*	5.26 ± 2.02	5.55 ± 0.78	7.29 ± 2.54			0.001**
**Bladder JOA**							
**Preoperative**	*Med ± STD*	-0.14 ± 0.74	-0.1 ± 0.66	-0.13 ± 0.61	-0.12 ± 0.69		***0.792***
**Postoperative**	*Med ± STD*	-0.05 ± 0.37	-0.02 ± 0.27	0 ± 0	-0.03 ± 0.31		***0.739***
**Change ∆**	*Med ± STD*	0.09 ± 0.52	0.07 ± 0.46	0.13 ± 0.61			***0.884***

^b^One-Way ANOVA Test & Bonferroni Test

^e^Wilcoxon Signed Rank Test

**p < 0.01 *p < 0.05

A statistically significant difference was observed between the recovery rates of the patients by group (*p* = 0.001;*p* < 0.01). The recovery rate in the group 1 was significantly lower than that in the group 2 and group 3 (*p* = 0.001;*p* < 0.01, *p* = 0.001;*p* < 0.01). Recovery rates also significant differed by groups and the group 3 had a high rate of very good recovery, while the group 2 had a high rate of good recovery. (Table 4)

**Table 4:** Recovery rates of cases by groups

	Group 1 (*n* = 129)	Group2(*n* = 123)	Group 3 (*n* = 24)		^d^ *p*
**Total JOA Preoperative** **Postoperative**	12.1 ± 5.69	12.06 ± 5.62	11.57 ± 5.57	**0.** 001**	
	23.85 ± 5.05	25.19 ± 3.35	26.04 ± 3.35		
**Rate of Recovery (%)**	Med ± STD	69.31 ± 17.82	77.54 ± 10.61	82.67 ± 13.03	0.001**
	Median (Min–Max)	73.3(15.3–94.1)	78.6(42.8–94.1)	88.6(50–95)	
	**Very good**	7 (5.4)	12 (9.8)	9 (37.5)	0.001**
	**Good**	72 (55.8)	85 (69.1)	10 (41.7)	
	**Fair**	31 (24.0)	24 (19.5)	5 (20.8)	
	**Poor**	19 (14.7)	2 (1.6)	0	

^b^One-Way ANOVA Test & Bonferroni Test

^e^Wilcoxon Signed Rank Test

**p < 0.01 *p < 0.05

In Group 1, the complication rate was 1.6%, consisting of two cases of postoperative wound infection. In Group 2, the overall complication rate was 3.3%, including three wound infections (2.4%) and one reoperation due to screw malposition (0.8%). In Group 3, the complication rate was 4.2%, with one case of cage dislocation requiring revision. No dural injury or spondylodiscitis was observed in any group.

## Discussion

Recurrent lumbar disc herniation can be successfully treated with repeated microdiscectomy with or without fusion. Previous studies have compared revision microdiscectomy alone to discectomy with fusion in the literature [6]. However, there is no consensus on the choice of surgical technique.

Revision microdiscectomy alone as a treatment for recurrent disc herniation has been reported in a previous studies and has been reported to be relatively simple and effective, providing good results but with a relative risk of recurrence and instability [10]. Given these risks, some surgeons recommend fusion surgery to prevent recurrence and instability [6]. Nevertheless, additional risks, including loss of mobile segment, pseudoarthrosis, and adjacent segment disease, should also be considered with the addition of fusion [11].

Age has been identified as a significant factor influencing the recurrence of lumbar disc herniation, with varying trends reported in the literature. Yao et al. reported that advanced age increased the likelihood of reherniation [12]. Although Kosztowski et al. stated that recurrence was more prevalent under the age of 50 years due to a more active period, in the present study, the mean age at which recurrence surgery was performed was 54.14 ± 11.88 [13]. Jansson et al. reported a higher rate of recurrence < 40 years of age and a lower rate > 60 years of age [14]. L4-5 and L5-S1 levels were the most prevalent levels of recurrent disc herniation reported in the relevant literature [15]. In our series, disc recurrence was most prevalent at the L4-5 level.

In the present study, postoperative radicular and lumbar VAS scores were higher in the group 1(revision microdiscectomy) than those in the other groups. In a similar study, Ahsan et al. reported higher postoperative VAS scores in the revision microdiscectomy compared to those in patients who underwent fusion [8]. Gallal et al. found that the lumbar VAS scores were high in the revision microdiscectomy group. The recovery rates based on VAS scores were 71.8% in the group 1, 75.5% in the group 2, and 77% in the group 3 in the present study. Mashhadinezhad et al. reported a recovery rate of 77.4% in overall postoperative VAS score (VAS Back 73% and VAS Leg 81.8%) [7]. Ahsan et al. also reported that the recovery based on VAS scores was 70.5% in the revision microdiscectomy group and 83.5% in the discectomy + PLF + TLIF group [8]. In the light of the aforementioned studies, transpedicular stabilization with revision microdiscectomy reduced low back pain, leg pain, and sciatic pain. We suggest that it would be more appropriate to perform fusion procedures in the preoperative period, especially in patients with high VAS scores.

Both posterolateral fusion with transpedicular screws and interbody fusion with PLIF systems can be performed in fusion cases. A comparison with the results reported in the relevant literature showed that patients who underwent fusion were usually operated with transpedicular screws and the PLIF system. Interbody fusion was preferred to preserve disc height and root-canal width [16]. In the present study, we separately evaluated these two fusion groups. In this study, postoperative JOA scores were higher in the group 3 than in the group 2. However, this difference was not statistically significant except for the SLR test.

A comparison of the group 2 and group 3 indicated that the preoperative total JOA scores increased from 12.1 ± 5.69 and 11.57 ± 5.57 to 23.85 ± 5.05 and 26.04 ± 3.35, respectively. These results were consistent with those reported by Ahsan et al. [8]. Fu et al. compared the microdiscectomy and discectomy + PLF groups and found that the total JOA scores were 25.3 ± 4.7 and 25.6 ± 4.7, respectively [9]. Similarly, Galal et al. found that the postoperative total JOA scores of the two groups were similar [11]. A general assessment indicates that the scores were higher after fusion, regardless of whether there was a statistical difference. On the basis of the above results, it was observed that patients were less painful and more functional after recovery of certain complaints, including the feeling of heaviness in the low back in the near postoperative period, compared to revision microdiscectomy.

JOA recovery scale is a widely used clinical assessment method following spinal surgery. In a study by Fu et al., the recovery rate was 78.3% in the revision microdiscectomy group and 83.3% in the discectomy + PLF group [9]. In a systematic review by Dower et al., which investigated 24 studies, the recovery rate was 70.8% in the revision microdiscectomy group and 86.6% in the discectomy + PLF group [17]. In the present study, these rates were 69.31% and 77.54%, respectively. Upon literature review, there were also studies that reported recovery rates in the revision microdiscectomy group of 90% [6, 18]. In a study by Lee et al., the success rate decreased to 45% [19]. The discectomy + PLF group was one step ahead in terms of both pain and functional well-being, as shown in previous assessment scales.

This study has several limitations. It retrospectively analyzed patients with recurrent lumbar disc herniation. Due to the study’s design, the surgical approach for the three groups was not randomized but was instead based on the physician’s clinical experience. Consequently, larger prospective studies are needed to yield more objective results. Since treatment decisions relied solely on the surgeon’s judgment, selection bias in the choice of surgical method cannot be ruled out.

## Conclusion

This study demonstrated that both revision microdiscectomy and discectomy with fusion yield successful outcomes for the surgical treatment of recurrent lumbar disc herniation. Revision microdiscectomy is typically preferred for younger patients, especially those without significant low back pain, whereas fusion is more commonly performed in older patients and those with predominant low back pain. Postoperative pain control can be challenging following revision microdiscectomy, while fusion procedures carry the risk of complications associated with instrumentation. These findings suggest that fusion may be more suitable for patients of advanced age and those with higher VAS scores. Complications associated with instrumentation in fusion procedures can be observed. Ultimately, the choice of surgical approach should be carefully tailored to the patient’s individual characteristics. Further prospective studies are needed to provide stronger evidence on the optimal treatment approach for different patient profiles.

## Electronic supplementary material

Below is the link to the electronic supplementary material.

Supplementary Material 1

Supplementary Material 2

Supplementary Material 3

## Data Availability

No datasets were generated or analysed during the current study.
