# Supplementary figures and images for: Comparison of discectomy with and without fusion in the surgical treatment of recurrent lumbar disc herniation
Source: Neurosurg Rev. 2025 Jul 5;48(1):542. doi: 10.1007/s10143-025-03687-8 (PMC12227494; doi:10.1007/s10143-025-03687-8)

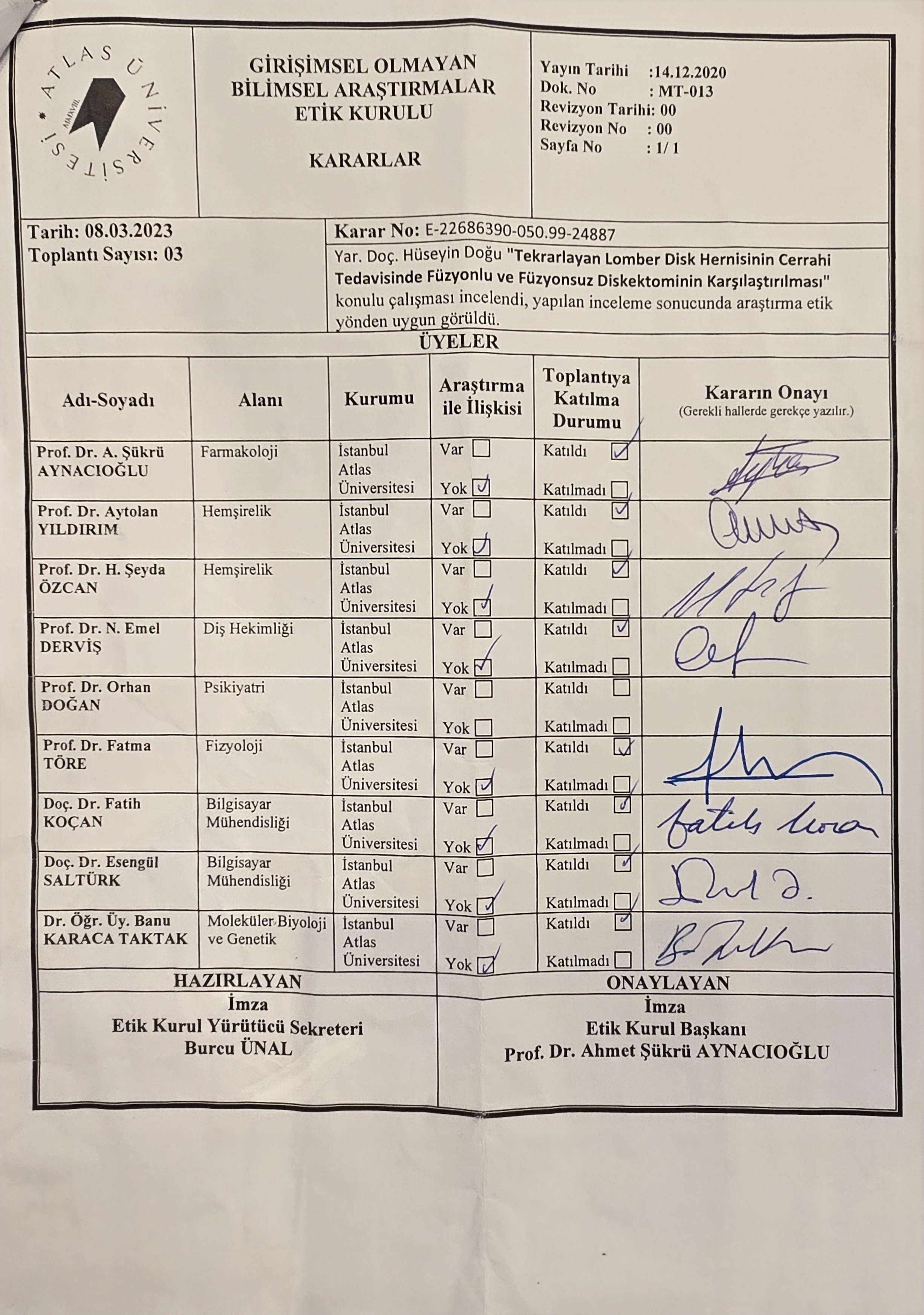

Supplement: Supplementary file 1 — Supplementary Material 1 [file 10143_2025_3687_MOESM1_ESM.jpg]

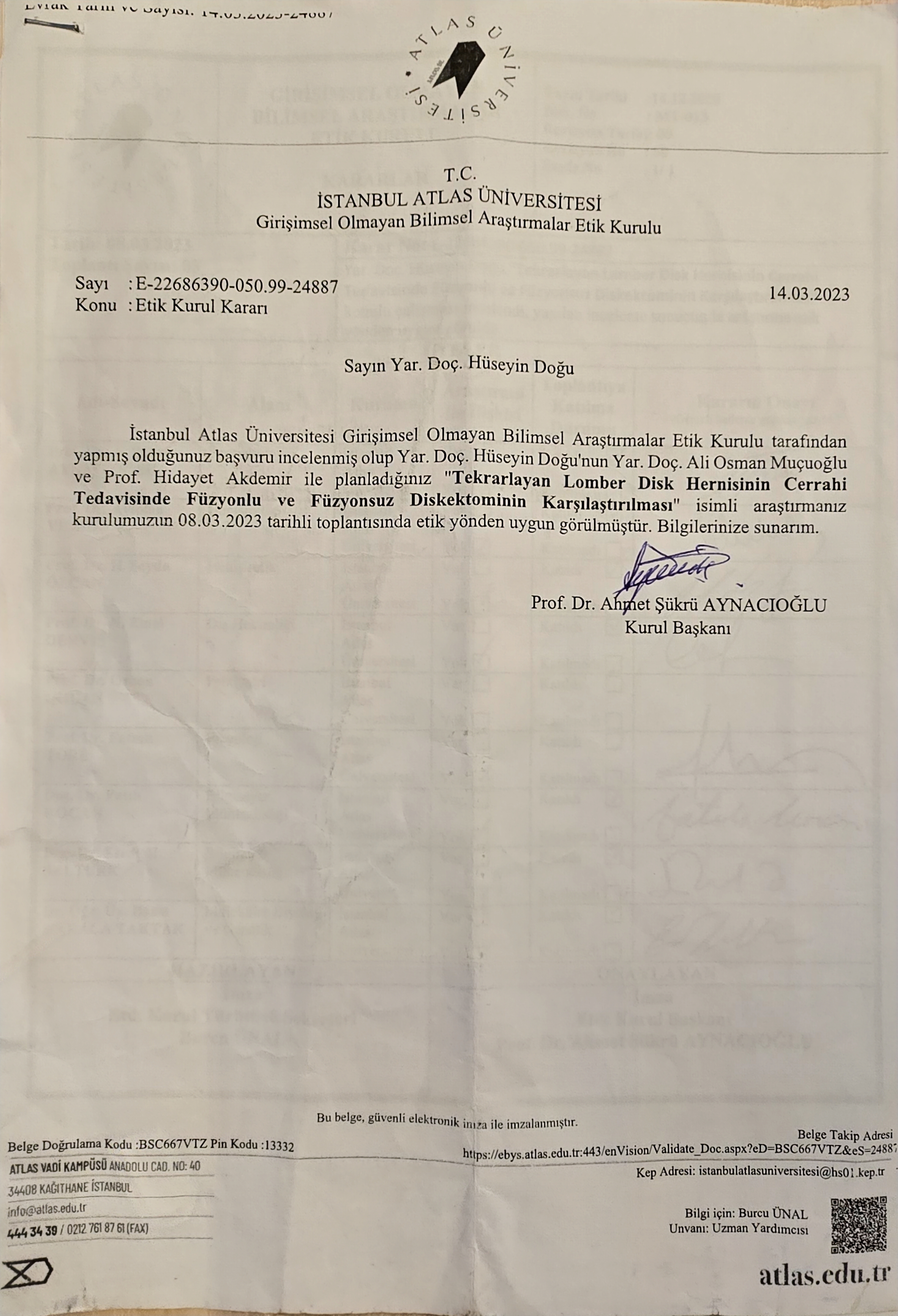

Supplement: Supplementary file 2 — Supplementary Material 2 [file 10143_2025_3687_MOESM2_ESM.jpg]
