# Supplementary material for: Comparison of discectomy with and without fusion in the surgical treatment of recurrent lumbar disc herniation
Source: Neurosurg Rev. 2025 Jul 5;48(1):542. doi: 10.1007/s10143-025-03687-8 (PMC12227494; doi:10.1007/s10143-025-03687-8)
Supplement: Supplementary file 3 — Supplementary Material 3 [file 10143_2025_3687_MOESM3_ESM.docx]

**BULGULAR**

Araştırma… tarihleri arasında … Hastanesinde %43,5’i (n=120) erkek, %56,5’i (n=156) kadın olmak üzere toplam 276 olguyla yapılmıştır. Olguların yaşları 28 ile 87 arasında değişmekte olup; ortalaması 54,14±11,88’dir.

**Tablo 1: Tanımlayıcı Özelliklerin Dağılımları**

|  |  | **n (%)** |
| --- | --- | --- |
| **Cinsiyet** | **Erkek** | 120 (43,5) |
|  | **Kadın** | 156 (56,5) |
| **Yaş** | *Ort±Ss* | 54,14±11,88 |
|  | *Medyan (Min-Maks)* | 54 (28-87) |
| **Taraf** | **Sol** | 140 (50,7) |
|  | **Sağ** | 136 (49,3) |
| **Seviye** | **L1-L2** | 2 (0,7) |
|  | **L2-L3** | 1 (0,4) |
|  | **L3-L4** | 22 (8,0) |
|  | **L4-L5** | 167 (60,5) |
|  | **L5-S1** | 84 (30,4) |
| **Nüks (ay)** | *Ort±Ss* | 30,49±21,14 |
|  | *Medyan (Min-Maks)* | 24 (6-121) |
| **Grup** | **LDH+LDH** | 129 (46,7) |
|  | **LDH+PSE** | 123 (44,6) |
|  | **LDH+PSE+TLIF** | 24 (8,7) |

Araştırmaya katılan olguların %50,7’sinin (n=140) tarafları sol, %49,3’ü (n=136) sağ olduğu görülmüştür.

Olguların seviyeleri incelendiğinde; %0,7’sinin (n=2) L1-L2, %0,4’ü (n=1) L2-L3, %8’inin (n=22) L3-L4, %60,5’inin (n=167) L4-L5, %30,4’ünün (n=84) L5-S1 olduğu görülmüştür.

Olguların nüks zamanları 6 ile 121 ay arasında değişmekte olup; ortalama süre 30,49±21,14 aydır.

Araştırmaya katılan olguların grupları incelendiğinde; %46,7’sinin (n=129) LDH, %44,6’sının (n=123) LDH+PSE, %8,7’sinin (n=24) LDH+PSE+TLIF gruplarında oldukları görülmüştür.

**Tablo 2: Gruplara Göre Tanımlayıcı Özelliklerin Karşılaştırılması**

|  |  | **LDH+LDH (n=129)** | **LDH+PSE (n=123)** | **LDH+PSE+TLIF (n=24)** | ***p*** |
| --- | --- | --- | --- | --- | --- |
| **Cinsiyet** | **Erkek** | 68 (52,7) | 43 (35,0) | 9 (37,5) | ***^a^0,015**** |
|  | **Kadın** | 61 (47,3) | 80 (65,0) | 15 (62,5) |  |
| **Yaş** | *Ort±Ss* | 53,77±12,17 | 55,80±11,22 | 47,71±11,74 | ***^b^0,008***** |
|  | *Medyan (Min-Maks)* | 52 (28-87) | 56 (30-86) | 48 (30-68) |  |
| **Taraf** | **Sol** | 70 (54,3) | 58 (47,2) | 12 (50) | ***^a^0,528*** |
|  | **Sağ** | 59 (45,7) | 65 (52,8) | 12 (50) |  |
| **Seviye** | **L1-L2** | 1 (0,8) | 1 (0,8) | 0 (0) | ***^c^0,005***** |
|  | **L2-L3** | 0 (0) | 1 (0,8) | 0 (0) |  |
|  | **L3-L4** | 10 (7,8) | 12 (9,8) | 0 (0) |  |
|  | **L4-L5** | 66 (51,2) | 86 (69,9) | 15 (62,5) |  |
|  | **L5-S1** | 52 (40,3) | 23 (18,7) | 9 (37,5) |  |
| **Nüks (ay)** | *Ort±Ss* | 28,79±19,37 | 33,87±23,82 | 22,25±9,71 | ***^b^0,022**** |
|  | *Medyan (Min-Maks)* | 24 (6-90) | 25 (7-121) | 24 (6-38) |  |

*^a^Pearson Chi-Square Test*

*^b^One-Way ANOVA Test & Bonferroni Test*

*^c^Fisher Freeman Halton Test*

***p<0,01 *p<0,05*

Cinsiyetlerine göre olguların grupları arasında istatistiksel olarak anlamlı farklılık saptanmıştır (p=0,015; p<0,05). Erkeklerin LDH+LDH grubunda olma oranı, LDH+PSE grubunda olmasından daha fazladır. Kadınların LDH+PSE grubunda olma oranı, LDH+LDH grubunda olmasından daha fazladır.

Gruplara göre olguların yaşları arasında istatistiksel olarak anlamlı farklılık saptanmıştır (p=0,008; p<0,01). Farklılığın kaynağını belirlemek amacıyla yapılan ikili karşılaştırmalar neticesinde; LDH+PSE grubundakilerin yaşları, LDH+PSE+TLIF grubundakilerden daha fazladır (p=0,007; p<0,01).

Gruplara göre olguların tarafları arasında istatistiksel olarak anlamlı farklılık saptanmamıştır (p>0,05).

Gruplara göre olguların seviyeleri arasında istatistiksel olarak anlamlı farklılık saptanmıştır (p=0,005; p<0,01). LDH+PSE grubundakilerin seviyelerinin L4-L5 olma oranı, LDH+LDH grubunda olmasından daha fazlayken; seviyesi L5-S1 olma oranı daha azdır.

Gruplara göre olguların nüks süreleri arasında istatistiksel olarak anlamlı farklılık saptanmıştır (p=0,022; p<0,05). Farklılığın kaynağını belirlemek amacıyla yapılan ikili karşılaştırmalar neticesinde; LDH+PSE grubundakilerin nüks süreleri LDH+PSE+TLIF grubundakilerden anlamlı yüksektir (p=0,040; p<0,05).

**Tablo 3: Gruplara Göre Olgulara İlişkin Özelliklerin Dağılımları**

|  | | **LDH+LDH (n=129)** | **LDH+PSE (n=123)** | **LDH+PSE+TLIF (n=24)** | **Toplam** | ***^d^p*** |
| --- | --- | --- | --- | --- | --- | --- |
| **Radiküler VAS** | |  |  |  |  |  |
| **Preop** | *Ort±Ss* | 7,53±1,11 | 7,56±1,12 | 7,21±1,18 | 7,52±1,12 | ***0,379*** |
|  | *Medyan (Min-Maks)* | 8 (5-9) | 8 (5-9) | 7,5 (5-9) | 8 (5-9) |  |
| **Postop** | *Ort±Ss* | 2,02±0,77 | 1,81±0,82 | 1,58±0,65 | 1,89±0,79 | ***0,008***** |
|  | *Medyan (Min-Maks)* | 2 (1-4) | 2 (1-4) | 1,5 (1-3) | 2 (1-4) |  |
|  | *p* | ***^e^0,001***** | ***^e^0,001***** | ***^e^0,001***** |  |  |
| **Değişim ∆** | *Ort±Ss* | -5,51±1,19 | -5,75±1,24 | -5,63±1,13 |  | ***0,374*** |
| **Bel VAS** | |  |  |  |  |  |
| **Preop** | *Ort±Ss* | 7,36±1,13 | 7,39±1,13 | 6,71±0,91 | 7,32±1,12 | ***0,009***** |
|  | *Medyan (Min-Maks)* | 8 (4-9) | 8 (4-9) | 7 (5-8) | 8 (4-9) |  |
| **Postop** | *Ort±Ss* | 2,16±0,70 | 1,78±0,68 | 1,58±0,65 | 1,94±0,72 | ***0,001***** |
|  | *Medyan (Min-Maks)* | 2 (1-4) | 2 (1-3) | 1,5 (1-3) | 2 (1-4) |  |
|  | *p* | ***^e^0,001***** | ***^e^0,001***** | ***^e^0,001***** |  |  |
| **Değişim ∆** | *Ort±Ss* | -5,20±1,18 | -5,61±1,23 | -5,13±0,80 |  | ***0,019**** |

*^d^Kruskal Wallis Test & Dunn-Bonferroni Test*

*^e^Wilcoxon Signed Rank Test*

***p<0,01 *p<0,05*

***Radiküler VAS ölçümleri için;***

Gruplara göre olguların preop radiküler VAS skorları arasında istatistiksel olarak anlamlı farklılık saptanmamıştır (p>0,05).

Gruplara göre olguların postop radiküler VAS skorları arasında istatistiksel olarak anlamlı farklılık saptanmıştır (p=0,008; p<0,01). Farklılığın kaynağını belirlemek amacıyla yapılan ikili karşılaştırmalar neticesinde; LDH+LDH grubundakilerin postop radiküler VAS skorları, LDH+PSE ve LDH+PSE+TLIF grubundakilerden anlamlı seviyede yüksek saptanmıştır (p=0,047; p=0,030; p<0,05).

**LDH+LDH grubundaki** olguların preopa göre postop radiküler VAS skorlarındaki ortalama 5,51±1,19 birimlik düşüş istatistiksel olarak anlamlı saptanmıştır (p=0,001; p<0,01).

**LDH+PSE grubundaki** olguların preopa göre postop radiküler VAS skorlarındaki ortalama 5,75±1,24 birimlik düşüş istatistiksel olarak anlamlı saptanmıştır (p=0,001; p<0,01).

**LDH+PSE+TLIF grubundaki** olguların preopa göre postop radiküler VAS skorlarındaki ortalama 5,63±1,13 birimlik düşüş istatistiksel olarak anlamlı saptanmıştır (p=0,001; p<0,01).

Gruplara göre olguların preopa göre postop radiküler VAS skorlarındaki değişim miktarları arasında istatistiksel olarak anlamlı farklılık saptanmamıştır (p>0,05).

***Şekil 1: Radiküler VAS skorlarının değişimleri***

***Bel VAS ölçümleri için;***

Gruplara göre olguların preop bel VAS skorları arasında istatistiksel olarak anlamlı farklılık saptanmıştır (p=0,009; p<0,01). Farklılığın kaynağını belirlemek amacıyla yapılan ikili karşılaştırmalar neticesinde; LDH+PSE+TLIF grubundakilerin preop bel VAS skorları, LDH+LDH ve LDH+PSE grubundakilerden anlamlı seviyede düşük saptanmıştır (p=0,012; p=0,009; p<0,05).

Gruplara göre olguların postop bel VAS skorları arasında istatistiksel olarak anlamlı farklılık saptanmıştır (p=0,001; p<0,01). Farklılığın kaynağını belirlemek amacıyla yapılan ikili karşılaştırmalar neticesinde; LDH+LDH grubundakilerin postop bel VAS skorları, LDH+PSE ve LDH+PSE+TLIF grubundakilerden anlamlı seviyede yüksek saptanmıştır (p=0,001; p=0,001; p<0,01).

**LDH+LDH grubundaki** olguların preopa göre postop bel VAS skorlarındaki ortalama 5,20±1,18 birimlik düşüş istatistiksel olarak anlamlı saptanmıştır (p=0,001; p<0,01).

**LDH+PSE grubundaki** olguların preopa göre postop bel VAS skorlarındaki ortalama 5,61±1,23 birimlik düşüş istatistiksel olarak anlamlı saptanmıştır (p=0,001; p<0,01).

**LDH+PSE+TLIF grubundaki** olguların preopa göre postop bel VAS skorlarındaki ortalama 5,13±0,80 birimlik düşüş istatistiksel olarak anlamlı saptanmıştır (p=0,001; p<0,01).

Gruplara göre olguların preopa göre postop bel VAS skorlarındaki değişim miktarları arasında istatistiksel olarak anlamlı farklılık saptanmıştır (p=0,019; p<0,05). Farklılığın kaynağını belirlemek amacıyla yapılan ikili karşılaştırmalar neticesinde; LDH+LDH grubundakilerin değişim miktarları, LDH+PSE grubundakilerden anlamlı seviyede düşük saptanmıştır (p=0,044; p<0,05).

***Şekil 2: Bel VAS skorlarının değişimleri***

**Tablo 4: Gruplara Göre Olgulara İlişkin Özelliklerin Dağılımları**

|  | | **LDH+LDH (n=129)** | **LDH+PSE (n=123)** | **LDH+PSE+TLIF (n=24)** | **Toplam** | ***^d^p*** |
| --- | --- | --- | --- | --- | --- | --- |
| **Bel JOA** | |  |  |  |  |  |
| **Preop** | *Ort±Ss* | 1,52±0,67 | 1,52±0,68 | 1,5±0,78 | 1,52±0,68 | ***0,964*** |
|  | *Medyan (Min-Maks)* | 2 (0-3) | 2 (0-3) | 1,5 (0-3) | 2 (0-3) |  |
| **Postop** | *Ort±Ss* | 2,50±0,63 | 2,75±0,51 | 2,79±0,41 | 2,63±0,57 | ***0,001***** |
|  | *Medyan (Min-Maks)* | 3 (1-3) | 3 (1-3) | 3 (2-3) | 3 (1-3) |  |
|  | *p* | ***^e^0,001***** | ***^e^0,001***** | ***^e^0,001***** |  |  |
| **Değişim ∆** | *Ort±Ss* | 0,98±0,90 | 1,23±0,79 | 1,29±0,81 |  | ***0,071*** |
| **Bacak JOA** | |  |  |  |  |  |
| **Preop** | *Ort±Ss* | 1,25±0,69 | 1,24±0,69 | 1,21±0,72 | 1,24±0,69 | ***0,971*** |
|  | *Medyan (Min-Maks)* | 1 (0-2) | 1 (0-2) | 1 (0-2) | 1 (0-2) |  |
| **Postop** | *Ort±Ss* | 2,51±0,60 | 2,80±0,44 | 2,79±0,41 | 2,66±0,54 | ***0,001***** |
|  | *Medyan (Min-Maks)* | 3 (1-3) | 3 (1-3) | 3 (2-3) | 3 (1-3) |  |
|  | *p* | ***^e^0,001***** | ***^e^0,001***** | ***^e^0,001***** |  |  |
| **Değişim ∆** | *Ort±Ss* | 1,26±0,87 | 1,56±0,84 | 1,58±0,93 |  | ***0,040**** |
| **Yürüme JOA** | |  |  |  |  |  |
| **Preop** | *Ort±Ss* | 0,63±0,49 | 0,62±0,49 | 0,54±0,51 | 0,62±0,49 | ***0,727*** |
|  | *Medyan (Min-Maks)* | 1 (0-1) | 1 (0-1) | 1 (0-1) | 1 (0-1) |  |
| **Postop** | *Ort±Ss* | 2,62±0,56 | 2,7±0,54 | 2,71±0,55 | 2,66±0,55 | ***0,334*** |
|  | *Medyan (Min-Maks)* | 3 (0-3) | 3 (1-3) | 3 (1-3) | 3 (0-3) |  |
|  | *p* | ***^e^0,001***** | ***^e^0,001***** | ***^e^0,001***** |  |  |
| **Değişim ∆** | *Ort±Ss* | 1,99±0,72 | 2,08±0,75 | 2,17±0,76 |  | ***0,423*** |
| **DBK JOA** | |  |  |  |  |  |
| **Preop** | *Ort±Ss* | 0,68±0,6 | 0,67±0,61 | 0,75±0,68 | 0,68±0,61 | ***0,898*** |
|  | *Medyan (Min-Maks)* | 1 (0-2) | 1 (0-2) | 1 (0-2) | 1 (0-2) |  |
| **Postop** | *Ort±Ss* | 1,66±0,52 | 1,95±0,22 | 1,88±0,34 | 1,81±0,42 | ***0,001***** |
|  | *Medyan (Min-Maks)* | 2 (0-2) | 2 (1-2) | 2 (1-2) | 2 (0-2) |  |
|  | *p* | ***^e^0,001***** | ***^e^0,001***** | ***^e^0,001***** |  |  |
| **Değişim ∆** | *Ort±Ss* | 0,98±0,80 | 1,28±0,66 | 1,13±0,74 |  | ***0,012**** |

*^b^One-Way ANOVA Test & Bonferroni Test*

*^e^Wilcoxon Signed Rank Test*

***p<0,01 *p<0,05*

***Bel JOA ölçümleri için;***

Gruplara göre olguların preop bel JOA skorları arasında istatistiksel olarak anlamlı farklılık saptanmamıştır (p>0,05).

Gruplara göre olguların postop bel JOA skorları arasında istatistiksel olarak anlamlı farklılık saptanmıştır (p=0,001; p<0,01). Farklılığın kaynağını belirlemek amacıyla yapılan ikili karşılaştırmalar neticesinde; LDH+LDH grubundakilerin postop bel JOA skorları, LDH+PSE ve LDH+PSE+TLIF grubundakilerden anlamlı seviyede düşük saptanmıştır (p=0,001; p=0,023; p<0,01).

**LDH+LDH grubundaki** olguların preopa göre postop bel JOA skorlarındaki ortalama 0,98±0,90 birimlik artış istatistiksel olarak anlamlı saptanmıştır (p=0,001; p<0,01).

**LDH+PSE grubundaki** olguların preopa göre postop bel JOA skorlarındaki ortalama 1,23±0,79 birimlik artış istatistiksel olarak anlamlı saptanmıştır (p=0,001; p<0,01).

**LDH+PSE+TLIF grubundaki** olguların preopa göre postop bel JOA skorlarındaki ortalama 1,29±0,81 birimlik artış istatistiksel olarak anlamlı saptanmıştır (p=0,001; p<0,01).

Gruplara göre olguların preopa göre postop bel JOA skorlarındaki değişim miktarları arasında istatistiksel olarak anlamlı farklılık saptanmamıştır (p>0,05).

***Şekil 3: Bel JOA skorlarının değişimleri***

***Bacak JOA ölçümleri için;***

Gruplara göre olguların preop bacak JOA skorları arasında istatistiksel olarak anlamlı farklılık saptanmamıştır (p>0,05).

Gruplara göre olguların postop bacak JOA skorları arasında istatistiksel olarak anlamlı farklılık saptanmıştır (p=0,001; p<0,01). Farklılığın kaynağını belirlemek amacıyla yapılan ikili karşılaştırmalar neticesinde; LDH+LDH grubundakilerin postop bacak JOA skorları, LDH+PSE grubundakilerden anlamlı seviyede düşük saptanmıştır (p=0,001; p<0,01).

**LDH+LDH grubundaki** olguların preopa göre postop bacak JOA skorlarındaki ortalama 1,26±0,87 birimlik artış istatistiksel olarak anlamlı saptanmıştır (p=0,001; p<0,01).

**LDH+PSE grubundaki** olguların preopa göre postop bacak JOA skorlarındaki ortalama 1,56±0,84 birimlik artış istatistiksel olarak anlamlı saptanmıştır (p=0,001; p<0,01).

**LDH+PSE+TLIF grubundaki** olguların preopa göre postop bacak JOA skorlarındaki ortalama 1,58±0,93 birimlik artış istatistiksel olarak anlamlı saptanmıştır (p=0,001; p<0,01).

Gruplara göre olguların preopa göre postop bacak JOA skorlarındaki değişim miktarları arasında istatistiksel olarak anlamlı farklılık saptanmıştır (p=0,040; p<0,05). Farklılığın kaynağını belirlemek amacıyla yapılan ikili karşılaştırmalar neticesinde; LDH+LDH grubundakilerin değişim miktarları, LDH+PSE grubundakilerden anlamlı seviyede düşük saptanmıştır (p=0,040; p<0,05).

***Şekil 4: Bacak JOA skorlarının değişimleri***

***Yürüme JOA ölçümleri için;***

Gruplara göre olguların preop yürüme JOA skorları arasında istatistiksel olarak anlamlı farklılık saptanmamıştır (p>0,05).

Gruplara göre olguların postop yürüme JOA skorları arasında istatistiksel olarak anlamlı farklılık saptanmamıştır (p>0,05).

**LDH+LDH grubundaki** olguların preopa göre postop yürüme JOA skorlarındaki ortalama 1,99±0,72 birimlik artış istatistiksel olarak anlamlı saptanmıştır (p=0,001; p<0,01).

**LDH+PSE grubundaki** olguların preopa göre postop yürüme JOA skorlarındaki ortalama 2,08±0,75 birimlik artış istatistiksel olarak anlamlı saptanmıştır (p=0,001; p<0,01).

**LDH+PSE+TLIF grubundaki** olguların preopa göre postop yürüme JOA skorlarındaki ortalama 2,17±0,76 birimlik artış istatistiksel olarak anlamlı saptanmıştır (p=0,001; p<0,01).

Gruplara göre olguların preopa göre postop yürüme JOA skorlarındaki değişim miktarları arasında istatistiksel olarak anlamlı farklılık saptanmamıştır (p>0,05).

***Şekil 5: Yürüme JOA skorlarının değişimleri***

***DBK JOA ölçümleri için;***

Gruplara göre olguların preop DBK JOA skorları arasında istatistiksel olarak anlamlı farklılık saptanmamıştır (p>0,05).

Gruplara göre olguların postop DBK JOA skorları arasında istatistiksel olarak anlamlı farklılık saptanmıştır (p=0,001; p<0,01). Farklılığın kaynağını belirlemek amacıyla yapılan ikili karşılaştırmalar neticesinde; LDH+LDH grubundakilerin postop bel JOA skorları, LDH+PSE ve LDH+PSE+TLIF grubundakilerden anlamlı seviyede düşük saptanmıştır (p=0,001; p=0,023; p<0,01).

**LDH+LDH grubundaki** olguların preopa göre postop DBK JOA skorlarındaki ortalama 0,98±0,80 birimlik artış istatistiksel olarak anlamlı saptanmıştır (p=0,001; p<0,01).

**LDH+PSE grubundaki** olguların preopa göre postop DBK JOA skorlarındaki ortalama 1,28±0,66 birimlik artış istatistiksel olarak anlamlı saptanmıştır (p=0,001; p<0,01).

**LDH+PSE+TLIF grubundaki** olguların preopa göre postop DBK JOA skorlarındaki ortalama 1,13±0,74 birimlik artış istatistiksel olarak anlamlı saptanmıştır (p=0,001; p<0,01).

Gruplara göre olguların preopa göre postop DBK JOA skorlarındaki değişim miktarları arasında istatistiksel olarak anlamlı farklılık saptanmıştır (p=0,012; p<0,05). Farklılığın kaynağını belirlemek amacıyla yapılan ikili karşılaştırmalar neticesinde; LDH+LDH grubundakilerin değişim miktarları, LDH+PSE grubundakilerden anlamlı seviyede düşük saptanmıştır (p=0,009; p<0,05).

***Şekil 6: DBK JOA skorlarının değişimleri***

**Tablo 5: Gruplara Göre Olgulara İlişkin Özelliklerin Dağılımları**

|  | | **LDH+LDH (n=129)** | **LDH+PSE (n=123)** | **LDH+PSE+TLIF (n=24)** | ***Toplam*** | ***^d^p*** |
| --- | --- | --- | --- | --- | --- | --- |
| **Duyu JOA** | |  |  |  |  |  |
| **Preop** | *Ort±Ss* | 0,76±0,6 | 0,73±0,59 | 0,75±0,68 | 0,75±0,6 | ***0,937*** |
|  | *Medyan (Min-Maks)* | 1 (0-2) | 1 (0-2) | 1 (0-2) | 1 (0-2) |  |
| **Postop** | *Ort±Ss* | 1,67±0,49 | 1,76±0,43 | 1,75±0,44 | 1,72±0,46 | ***0,317*** |
|  | *Medyan (Min-Maks)* | 2 (0-2) | 2 (1-2) | 2 (1-2) | 2 (0-2) |  |
|  | *p* | ***^e^0,001***** | ***^e^0,001***** | ***^e^0,001***** |  |  |
| **Değişim ∆** | *Ort±Ss* | 0,91±0,75 | 1,03±0,56 | 1,00±0,72 |  | ***0,588*** |
| **Motor JOA** | |  |  |  |  |  |
| **Preop** | *Ort±Ss* | 1,36±0,62 | 1,36±0,63 | 1,42±0,65 | 1,36±0,63 | ***0,879*** |
|  | *Medyan (Min-Maks)* | 1 (0-2) | 1 (0-2) | 1,5 (0-2) | 1 (0-2) |  |
| **Postop** | *Ort±Ss* | 1,68±0,48 | 1,67±0,47 | 1,71±0,46 | 1,68±0,48 | ***0,896*** |
|  | *Medyan (Min-Maks)* | 2 (0-2) | 2 (1-2) | 2 (1-2) | 2 (0-2) |  |
|  | *p* | ***^e^0,001***** | ***^e^0,001***** | ***^e^0,035**** |  |  |
| **Değişim ∆** | *Ort±Ss* | 0,33±0,80 | 0,31±0,53 | 0,29±0,62 |  | ***0,978*** |

*^b^One-Way ANOVA Test & Bonferroni Test*

*^e^Wilcoxon Signed Rank Test*

***p<0,01 *p<0,05*

***Duyu JOA ölçümleri için;***

Gruplara göre olguların preop duyu JOA skorları arasında istatistiksel olarak anlamlı farklılık saptanmamıştır (p>0,05).

Gruplara göre olguların postop duyu JOA skorları arasında istatistiksel olarak anlamlı farklılık saptanmamıştır (p>0,05).

**LDH+LDH grubundaki** olguların preopa göre postop duyu JOA skorlarındaki ortalama 0,91±0,75 birimlik artış istatistiksel olarak anlamlı saptanmıştır (p=0,001; p<0,01).

**LDH+PSE grubundaki** olguların preopa göre postop duyu JOA skorlarındaki ortalama 1,03±0,56 birimlik artış istatistiksel olarak anlamlı saptanmıştır (p=0,001; p<0,01).

**LDH+PSE+TLIF grubundaki** olguların preopa göre postop duyu JOA skorlarındaki ortalama 1,00±0,72 birimlik artış istatistiksel olarak anlamlı saptanmıştır (p=0,001; p<0,01).

Gruplara göre olguların preopa göre postop duyu JOA skorlarındaki değişim miktarları arasında istatistiksel olarak anlamlı farklılık saptanmamıştır (p>0,05).

***Şekil 7: Duyu JOA skorlarının değişimleri***

***Motor JOA ölçümleri için;***

Gruplara göre olguların preop motor JOA skorları arasında istatistiksel olarak anlamlı farklılık saptanmamıştır (p>0,05).

Gruplara göre olguların postop motor JOA skorları arasında istatistiksel olarak anlamlı farklılık saptanmamıştır (p>0,05).

**LDH+LDH grubundaki** olguların preopa göre postop motor JOA skorlarındaki ortalama 0,33±0,80 birimlik artış istatistiksel olarak anlamlı saptanmıştır (p=0,001; p<0,01).

**LDH+PSE grubundaki** olguların preopa göre postop motor JOA skorlarındaki ortalama 0,31±0,53 birimlik artış istatistiksel olarak anlamlı saptanmıştır (p=0,001; p<0,01).

**LDH+PSE+TLIF grubundaki** olguların preopa göre postop motor JOA skorlarındaki ortalama 0,29±0,62 birimlik artış istatistiksel olarak anlamlı saptanmıştır (p=0,035; p<0,05).

Gruplara göre olguların preopa göre postop motor JOA skorlarındaki değişim miktarları arasında istatistiksel olarak anlamlı farklılık saptanmamıştır (p>0,05).

***Şekil 8: Motor JOA skorlarının değişimleri***

**Tablo 6: Gruplara Göre JOA Skorlamalarının Değerlendirmeleri**

|  | | **LDH+LDH (n=129)** | **LDH+PSE (n=123)** | **LDH+PSE+TLIF (n=24)** | ***^d^p*** |
| --- | --- | --- | --- | --- | --- |
| **Subjektif Semptomlar JOA** | |  |  |  |  |
| **Preop** | *Ort±Ss* | 3,39±1,28 | 3,37±1,29 | 3,25±1,42 | ***0,881*** |
|  | *Medyan (Min-Maks)* | 3(0-6) | 3 (0-6) | 3 (1-6) |  |
| **Postop** | *Ort±Ss* | 7,63±1,06 | 8,25±1,03 | 8,29±0,81 | ***0,001***** |
|  | *Medyan (Min-Maks)* | 8 (3-9) | 8 (3-9) | 8 (6-9) |  |
|  | *p* | ***^e^0,001***** | ***^e^0,001***** | ***^e^0,001***** |  |
| **Değişim ∆** | *Ort±Ss* | 4,23±1,63 | 4,86±1,52 | 5,04±1,52 | ***0,002***** |
| **Klinik bulgular JOA** | |  |  |  |  |
| **Preop** | *Ort±Ss* | 2,79±1,21 | 2,76±1,22 | 2,92±1,41 | ***0,857*** |
|  | *Medyan (Min-Maks)* | 3 (0-6) | 3 (0-6) | 3 (0-6) |  |
| **Postop** | *Ort±Ss* | 5,01±1,25 | 5,38±0,73 | 5,33±0,82 | ***0,014**** |
|  | *Medyan (Min-Maks)* | 6 (2-6) | 6 (4-6) | 6 (4-6) |  |
|  | *p* | ***^e^0,001***** | ***^e^0,001***** | ***^e^0,001***** |  |
| **Değişim ∆** | *Ort±Ss* | 2,21±1,76 | 2,61±1,27 | 2,41±1,56 | ***0,121*** |
| **Aktivite JOA** | |  |  |  |  |
| **Preop** | *Ort±Ss* | 6,04±1,28 | 6,02±1,27 | 5,13±2,13 | ***0,088*** |
|  | *Medyan (Min-Maks)* | 6 (2-9) | 6 (2-9) | 5 (1-9) |  |
| **Postop** | *Ort±Ss* | 11,29±1,4 | 11,58±1,32 | 12,42±1,72 | ***0,065*** |
|  | *Medyan (Min-Maks)* | 12 (7-14) | 12 (8-14) | 13 (7-14) |  |
|  | *p* | ***^e^0,001***** | ***^e^0,001***** | ***^e^0,001***** |  |
| **Değişim ∆** | *Ort±Ss* | 5,26±2,02 | 5,55±0,78 | 7,29±2,54 | ***0,001***** |
| **Mesane JOA** | |  |  |  |  |
| **Preop** | *Ort±Ss* | -0,14±0,74 | -0,1±0,66 | -0,13±0,61 | ***0,792*** |
|  | *Medyan (Min-Maks)* | 0 (-6-0) | 0 (-6-0) | 0 (-3-0) |  |
| **Postop** | *Ort±Ss* | -0,05±0,37 | -0,02±0,27 | 0±0 | ***0,739*** |
|  | *Medyan (Min-Maks)* | 0 (-3-0) | 0 (-3-0) | 0 (0-0) |  |
|  | *p* | ***^e^0,046**** | ***^e^0,083*** | ***^e^0,317*** |  |
| **Değişim ∆** | *Ort±Ss* | 0,09±0,52 | 0,07±0,46 | 0,13±0,61 | ***0,884*** |
| **İyileşme Oranı (%)** | *Ort±Ss* | 69,31±17,82 | 77,54±10,61 | 82,67±13,03 | ***0,001***** |
|  | *Medyan (Min-Maks)* | 73,3(15,3-94,1) | 78,6(42,8-94,1) | 88,6(50-95) |  |
|  | **Çok iyi** | 7 (5,4) | 12 (9,8) | 9 (37,5) | ***0,001***** |
|  | **İyi** | 72 (55,8) | 85 (69,1) | 10 (41,7) |  |
|  | **Orta** | 31 (24,0) | 24 (19,5) | 5 (20,8) |  |
|  | **Kötü** | 19 (14,7) | 2 (1,6) | 0 |  |

*^b^One-Way ANOVA Test & Bonferroni Test*

*^e^Wilcoxon Signed Rank Test*

***p<0,01 *p<0,05*

***Subjektif Semptomlar JOA ölçümleri için;***

Gruplara göre olguların preop subjektif semptomlar JOA skorları arasında istatistiksel olarak anlamlı farklılık saptanmamıştır (p>0,05).

Gruplara göre olguların postop subjektif semptomlar JOA skorları arasında ise istatistiksel olarak anlamlı farklılık saptanmıştır (p=0,001; p<0,01). Anlamlılık incelendiğinde; LDH+LDH grubu skorları LDH+PSA ve LDH+PSE+TLİF gruplarından anlamlı düzeyde düşüktür (p=0,001; p=0,012; p<0,05). LDH+PSA ve LDH+PSE+TLİF gruplarının subjektif semptomlar JOA skorları arasında ise anlamlı farklılık saptanmamıştır (p>0,05).

**LDH+LDH grubundaki** olguların preopa göre postop subjektif semptomlar JOA skorlarındaki ortalama 4.23±1,63 birimlik artış istatistiksel olarak anlamlı saptanmıştır (p=0,001; p<0,01).

**LDH+PSE grubundaki** olguların preopa göre postop subjektif semptomlar JOA skorlarındaki ortalama 4,86±1,52 birimlik artış istatistiksel olarak anlamlı saptanmıştır (p=0,001; p<0,01).

**LDH+PSE+TLIF grubundaki** olguların preopa göre postop subjektif semptomlar JOA skorlarındaki ortalama 5.04±1.52 birimlik artış istatistiksel olarak anlamlı saptanmıştır (p=0,001; p<0,01).

Gruplara göre olguların preopa göre postop subjektif semptomlar JOA skorlarındaki değişim miktarları arasında istatistiksel olarak anlamlı farklılık saptanmıştır (p=0,002; p<0,01). Anlamlılık incelendiğinde LDH+LDH grubu skorlarının fark değerleri LDH+PSE+TLİF grubundan anlamlı düzeyde düşüktür (p=0,004; p<0,01). Diğer grupların ikili fark değerleri arasında anlamlılık saptanmamıştır (p>0,05).

***Şekil 7: Subjektif Semptomlar JOA skorlarının dağılımı***

***Klinik Bulgular JOA ölçümleri için;***

Gruplara göre olguların preop klinik bulgular JOA skorları arasında istatistiksel olarak anlamlı farklılık saptanmamıştır (p>0,05).

Gruplara göre olguların postop klinik bulgular JOA skorları arasında istatistiksel olarak anlamlı farklılık saptanmıştır (p=0,014; p<0,05). Anlamlılık incelendiğinde LDH+LDH grubu skorlarının fark değerleri LDH+PSEF grubundan anlamlı düzeyde düşüktür (p=0,013; p<0,05). Diğer grupların ikili klinik bulgular JOA değerleri arasında anlamlılık saptanmamıştır (p>0,05).

**LDH+LDH grubundaki** olguların preopa göre postop klinik bulgular JOA skorlarındaki ortalama 2,21±1,76 birimlik artış istatistiksel olarak anlamlı saptanmıştır (p=0,001; p<0,01).

**LDH+PSE grubundaki** olguların preopa göre postop klinik bulgular JOA skorlarındaki ortalama 2,61±1,27 birimlik artış istatistiksel olarak anlamlı saptanmıştır (p=0,001; p<0,01).

**LDH+PSE+TLIF grubundaki** olguların preopa göre postop klinik bulgular JOA skorlarındaki ortalama 2,41±1,56 birimlik artış istatistiksel olarak anlamlı saptanmıştır (p=0,001; p<0,01).

Gruplara göre olguların preopa göre postop klinik bulgular JOA skorlarındaki değişim miktarları arasında istatistiksel olarak anlamlı farklılık saptanmamıştır (p>0,05).

***Şekil 7klinik bulgular JOA skorlarının dağılımı***

***Aktivite JOA ölçümleri için;***

Gruplara göre olguların preop aktivite JOA skorları arasında istatistiksel olarak anlamlı farklılık saptanmamıştır (p>0,05).

Gruplara göre olguların postop aktivite JOA skorları arasında istatistiksel olarak anlamlı farklılık saptanmamıştır (p>0,05).

**LDH+LDH grubundaki** olguların preopa göre postop aktivite JOA skorlarındaki ortalama 5,26±2,02 birimlik artış istatistiksel olarak anlamlı saptanmıştır (p=0,001; p<0,01).

**LDH+PSE grubundaki** olguların preopa göre postop aktivite JOA skorlarındaki ortalama 5,55±0,78 birimlik artış istatistiksel olarak anlamlı saptanmıştır (p=0,001; p<0,01).

**LDH+PSE+TLIF grubundaki** olguların preopa göre postop aktivite JOA skorlarındaki ortalama 7,29±2,54 birimlik artış istatistiksel olarak anlamlı saptanmıştır (p=0,001; p<0,01).

Gruplara göre olguların preopa göre postop aktivite JOA skorlarındaki değişim miktarları arasında istatistiksel olarak anlamlı farklılık saptanmıştır (p=0,001; p<0,01). Farklılığın kaynağını belirlemek amacıyla yapılan ikili karşılaştırmalar neticesinde; LDH+ PSE+TLIF grubundakilerin değişim miktarları, LDH+LDH ve LDH+PSE grubundakilerden anlamlı seviyede yüksek saptanmıştır (p=0,001; p=0,001; p<0,01).

***Şekil 9: Aktivite JOA skorlarının dağılımı***

***Mesane JOA ölçümleri için;***

Gruplara göre olguların preop mesane JOA skorları arasında istatistiksel olarak anlamlı farklılık saptanmamıştır (p>0,05).

Gruplara göre olguların postop mesane JOA skorları arasında istatistiksel olarak anlamlı farklılık saptanmamıştır (p>0,05).

**LDH+LDH grubundaki** olguların preopa göre postop mesane JOA skorlarındaki ortalama 0,09±0,52 birimlik artış istatistiksel olarak anlamlı saptanmıştır (p=0,046; p<0,05).

**LDH+PSE grubundaki** olguların preopa göre postop mesane JOA skorlarındaki değişim istatistiksel olarak anlamlı saptanmamıştır (p>0,05).

**LDH+PSE+TLIF grubundaki** olguların preopa göre postop mesane JOA skorlarındaki değişim istatistiksel olarak anlamlı saptanmamıştır (p>0,05).

Gruplara göre olguların preopa göre postop mesane JOA skorlarındaki değişim miktarları arasında istatistiksel olarak anlamlı farklılık saptanmamıştır (p>0,05).

***Şekil 10: Mesane JOA skorlarının dağılımı***

Gruplara göre olguların iyileşme oranları arasında ise istatistiksel olarak anlamlı farklılık saptanmıştır (p=0,001; p<0,01). Anlamlılık incelendiğinde; LDH+LDH grubu oranları LDH+PSA ve LDH+PSE+TLİF gruplarından anlamlı düzeyde düşüktür (p=0,001; p=0,001; p<0,01). LDH+PSA ve LDH+PSE+TLİF gruplarının iyileşme oranları arasında ise anlamlı farklılık saptanmamıştır (p=0,352; p>0,05).


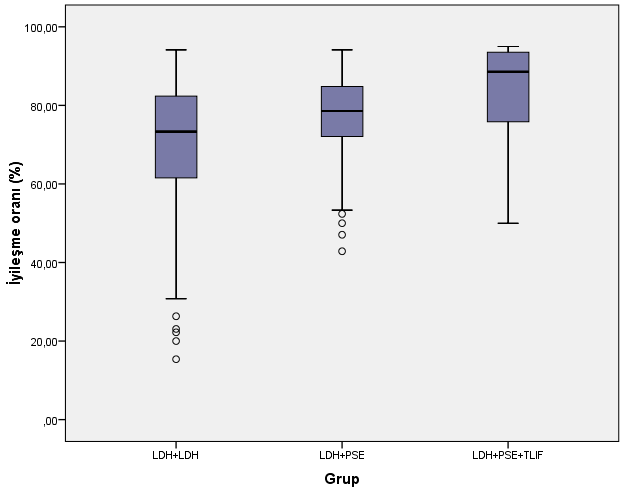


***Şekil : Gruplara göre iyileşme oranlarının dağılımı***

İyileşme oranları da gruplara göre anlamlı farklılık göstermiş olup LDH+PSE+TLİF grubun iyileşmede çok iyi sonuç alam oranı yüksek iken; LDH+PSE grubunun iyi düzeyde iyileşme oranı yüksek bulunmuştur.

***Şekil : Gruplara göre iyileşme sınıflamalarının dağılımı***

# İstatistiksel İncelemeler

Çalışmada elde edilen bulgular değerlendirilirken, istatistiksel analizler için SPSS 26 (***S****tatistical****P****ackage for the****S****ocial****S****ciences*) programı kullanıldı. Çalışma verileri değerlendirilirken, nicel değişkenler ortalama, standart sapma, medyan, min ve max değerleriyle, nitel değişkenler frekans ve yüzde gibi tanımlayıcı istatistiksel metodlar ile gösterildi. Verilerin normal dağılıma uygunluklarının değerlendirilmesinde Shapiro Wilks test ve Box Plot grafiklerden yararlanıldı.

Normal dağılım gösteren niceliksel iki grup değerlendirmelerinde Oneway Anova test ve farklılığa neden olan grubun tespitinde Bonferroni test kullanıldı.

Normal dağılım göstermeyen değişkenlerin grup içi değerlendirmelerde Wilcoxon Signed Rank test; üç grup ve üzeri karşılaştırmalarında Kruskal Wallis test ve farklılığa neden olan grubun tespitinde Dunn test kullanıldı.

Niteliksel verilerin karşılaştırılmasında ise Ki-Kare test, Fisher Freeman Halton Test kullanıldı.

Sonuçlar % 95’lik güven aralığında, anlamlılık p<0.05 düzeyinde değerlendirildi.
